# Wave scattering from graphene-covered circular dielectric wire collections analysed using the single-wire part inversion: diffraction radiation case

**DOI:** 10.1098/rsta.2024.0344

**Published:** 2025-08-14

**Authors:** Dariia O. Herasymova, Denys M. Natarov, Mario Lucido, Oleksandr I. Nosych, Sergii V. Dukhopelnykov

**Affiliations:** ^1^ Laboratory of Micro and Nano Optics, O Ya Usikov Institute for Radiophysics and Electronics National Academy of Sciences of Ukraine, Kharkiv, Ukraine; ^2^ Department of Electrical and Information Engineering “Maurizio Scarano”, University of Cassino and Southern Lazio, Cassino 03043, Italy; ^3^ 2EUt+ Institute of Nanomaterials and Nanotechnologies-EUTINN, European University of Technology; ^4^ Institut d’Electronique et de Technologies du numéRique, Universite de Rennes, Rennes, France

**Keywords:** diffraction radiation, graphene, electron beam, nanowires, grating, plasmon mode

## Abstract

We consider infrared (IR)-range diffraction radiation (DR) from finite configurations of circular graphene-covered dielectric nanowires excited by the density-modulated beam of charged particles. The beam velocity is assumed constant, and its field in the free space is considered as the incident one. The characterization of graphene employs the quantum-theory Kubo formalism and the resistive-sheet boundary conditions involving the frequency-dependent graphene surface impedance. To transform the problem into a well-conditioned algebraic equation for the field expansion coefficients, we use the separation of variables in the local coordinates and the addition theorem for the cylindrical functions. This leads to explicit inversion of the single-wire part of the problem, i.e. to the regularization, provides easy control of the accuracy and enables us to study fine resonance effects associated with the natural modes of the wire collections as open resonators.

This article is part of the theme issue ‘Analytically grounded full-wave methods for advances in computational electromagnetics’.

## 1. Introduction

Among the various types of radiation, linked to charged particles moving along straight-line trajectories, one may encounter Cherenkov radiation, transition radiation, diffraction radiation (DR) and Smith–Purcell radiation. Cherenkov radiation arises if a charged particle moves faster than the phase velocity of light in unbounded dielectric medium. Transition radiation results from a charged particle crossing a boundary between domains with different permittivities. In contrast, DR corresponds to the emission of electromagnetic waves if charged particles pass near dielectric or metal obstacles without penetrating or touching them [1–6].

Smith–Purcell radiation was predicted and then found for charged particles travelling near periodic structures [1,2]. Hence, this radiation can be identified as a special case of DR.

One of the key areas of practical use of DR is the non-invasive control of particle beams in accelerators and colliders using beam position monitors (BPMs) [7,8]. Today, this mature technology develops towards microscale and nanoscale devices and electromagnetic wave emission in the terahertz (THz) to ultraviolet ranges, using the resonances of high-refractive-index scatterers. Consequently, modelling of DR-based nano-optical BPMs is a timely and promising research subject.

An emerging and prospective strategy for the next generation of compact particle accelerators involves periodic dielectric gratings driven by a laser. Dielectric laser accelerators (DLAs) produce accelerating gradients two orders of magnitude higher than traditional microwave ones, achieved due to use of various gratings of several hundred circular silicon nanowires [9,10]. Clearly, the electron beams in DLAs emit short-wave DR, accompanied with various resonance phenomena. This makes electromagnetic analysis of DR effects on such gratings interesting and important.

Note that, there is an alternative to the natural modes of high-refractive index elements: plasmon modes supported by graphene-covered low-index scatterers. Recently, graphene has garnered significant attention due to its remarkable properties, such as charge carrier high mobility, controlled by DC bias [11,12]. Research efforts are presently concentrated on assessing the characteristics of graphene patterned into discs, tubes, strips and gratings [12–16]. A graphene sheet is capable of supporting a plasmon wave [11,14]. On patterned graphene, this wave reflects from the sample edges and forms standing waves, referred to as plasmon natural modes. Their frequencies depend on the sample size and are located in the THz and infrared (IR) ranges, respectively, for micro- and nano-sized graphene cavities.

In this work, we analyse the DR effect associated with the two-dimensional models of the THz/IR-range BPM built on two identical circular graphene-covered dielectric nanowires and the DLA section built as a finite array of them. Note that, such nanowires can be readily fabricated [17]. As usual in DR studies, we treat these problems as the classical wave-scattering problems, supplemented with the quantum-theory description of the graphene conductivity, where the incident wave is the free-space field of the harmonically modulated electron beam.

Although the scattering of waves from a collection of circular cylinders has been studied since the 1950s [18], reducing it to a numerical code that has a mathematically guaranteed accuracy is still neglected. Inspection of the preceding publications shows that the bulk of them ignore the divergence of the resulting discretized solutions [18–24]. Here, we explain how this defect can be fixed with the aid of the analytical inversion of the single-wire scattering. The trusted numerical results demonstrate various fine resonance phenomena.

This work builds up on the conference papers [25–27], which have been considerably deepened and extended.

## 2. DR problem formulation

Consider a flat zero-thickness electron beam, which moves parallel to the *x*-axis at distance *h* from the plate, with fixed velocity v=βc (β<1). Assume that the charge density is time-harmonically modulated with the cyclic frequency *ω* and hence has the form, $\rho =\rho_{0}\delta (y-h)e^{ikx/\beta }e^{-i\omega t}$, where δ (⋅) is the Dirac delta function, $\rho_{0}$ is the amplitude of modulation, k=ω/c is the free-space wavenumber and *c* is the light velocity.

As usual in the DR studies, we assume that the electron beam velocity and trajectory remain fixed, this is known as the *given-current approximation*. In this scenario, the incident wave is the field of the sheet current, i.e. it has the form of a slow inhomogeneous plane wave [6], the only component of the magnetic field of which is


(1.1) $$H_{z}^{0}\left(x,y\right)=A\beta \text{sign}\left(y-h\right)e^{-q|y-h|}e^{ikx/\beta },$$

where q=kγ/β, $\gamma ={\left(1-\beta^{2}\right)}^{1/2}$, sign(⋅)=±1, the time dependence $e^{-i\omega t}$ is omitted and $A=\rho_{0}c/2$ in SI units.

This is a surface wave sticking to the beam trajectory and decaying exponentially in the normal direction. It propagates with the same phase velocity as the beam particles. Note that, the field equation (1.1) is an antisymmetric function of the coordinate *y* and has a finite jump across the beam trajectory corresponding to the beam current. Note also that, equation (1.1) can be viewed as a Fourier transform of the field of the single charged wire-like particle [2].

**Figure 1.**
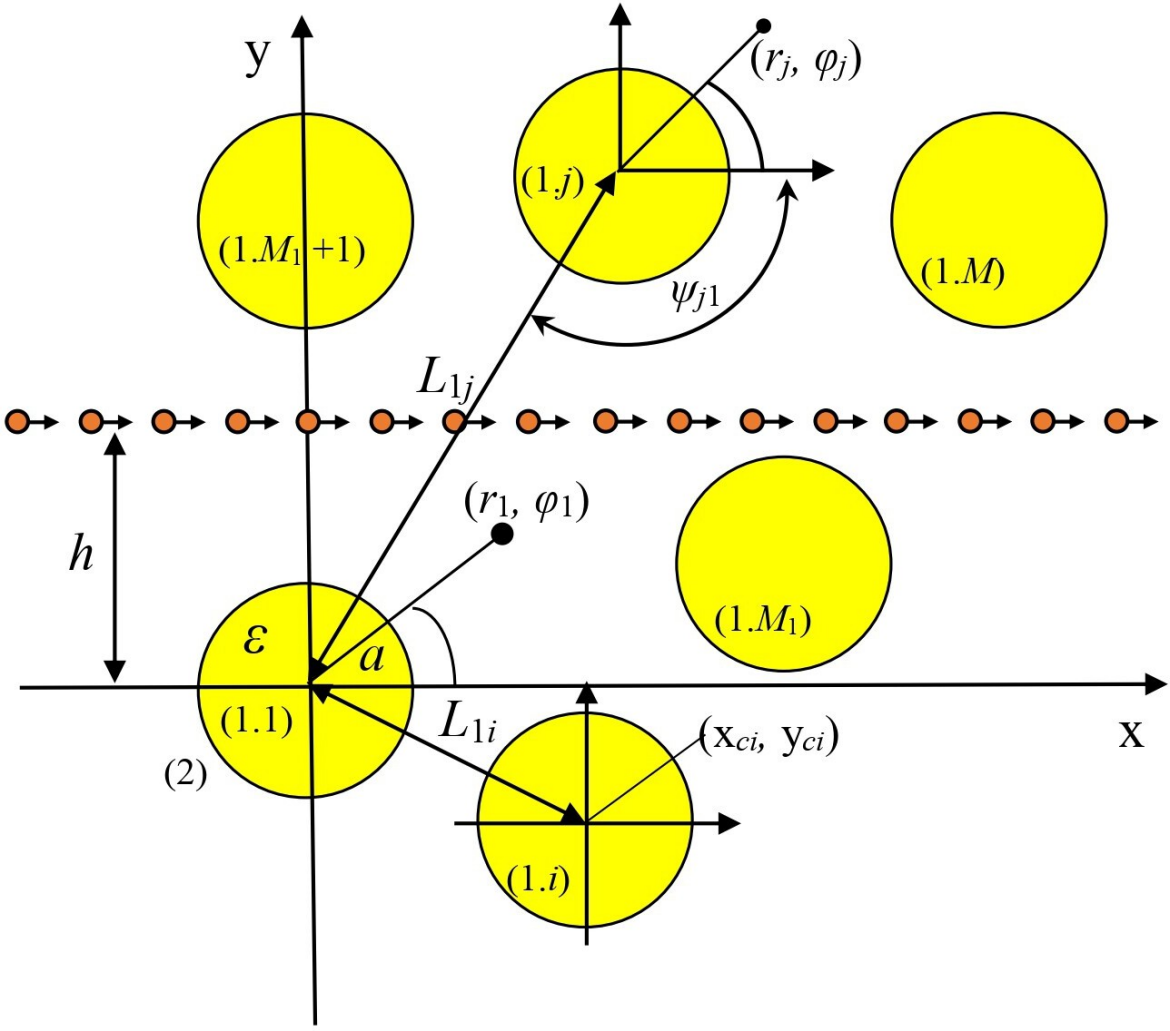
Cross-section of zero-thickness electron beam moving through *M* identical circular wires.

Thus, we are interested in the problem of the field equation (1.1) scattering from *M* identical circular dielectric wires with radius *a*, located in the free space as shown in figure 1, with their centres of cross-sections at (*x_p_*, *y_p_*). Let us denote the internal domain of the *p*th wire as region {1 .*p*}, and the domain, external to all wires as region {2}. Here, the wires located under the beam trajectory are numbered as *p* = 1, …, *M*_1_, in addition, those located above the beam trajectory are numbered as *p* = *M*_1_+1, …, *M*.

We introduce Cartesian and polar coordinates: global one with the origin at the first wire axis, $\vec{r}=(x,y)$, that is, x=rcos⁡φ, y=rsin⁡φ and $r=\sqrt{x^{2}+y^{2}},\;\varphi =\arctan(x/y)$, and *M* local ones with origins at each wire axis. The relative dielectric constant of the nanowires is denoted as $\varepsilon =\varepsilon^{\prime }+i\varepsilon^{\prime \prime }$, and all materials are non-magnetic. Then the refractive index of the wire material is $\alpha =\sqrt{\varepsilon }$.

Now the DR field problem can be viewed as a classical wave-scattering boundary value problem, where the incident field is the function (1.1). Such a problem consists of the Helmholtz equation with wavenumber *kα* in every {1 .*p*}, p=1,...,M, or *k* in {2}, the graphene-layer boundary conditions at the wire contours, i.e. at $r_{p}=a,\;\;0\leq \varphi_{p}<2\pi ,\;\;p=1,...M$, the Sommerfeld radiation condition at infinity, and last but not least, the condition of the local power finiteness. That is, the graphene-layer boundary conditions are the two equations,


(1.2) $$E_{\varphi_{p}}^{int\left(p\right)}-E_{\varphi_{p}}^{0}-E_{\varphi_{p}}^{ext}=0,\;\;E_{\varphi_{p}}^{int\left(p\right)}+E_{\varphi_{p}}^{0}+E_{\varphi_{p}}^{ext}=2ZZ_{0}\left[H_{,}^{int\left(p\right)}-H_{,}^{0}-H^{ext}\right],$$

where $E_{\varphi_{p}}^{0}$, $E_{\varphi_{p}}^{\mathrm{int}(p)}$ and $E_{\varphi_{p}}^{\text{ext}}$ are the incident, internal and external electric field *φ*-component, respectively, at the *p*th wire contour (similar notation hold for the magnetic field, *H* = *H_z_*),$Z_{0}=\sqrt{\mu_{0}/\varepsilon_{0}}$ is the free space impedance and *Z* is graphene surface impedance (explained further), normalized by *Z*_0_. Note that, these boundary conditions are two-side as they involve the limit values of the tangential field components from inside and outside of the contour. If *Z* = 0, they turn into the conventional PEC condition.

As known, the aforementioned set of conditions guarantees the solution uniqueness for all real-valued *k*, only if $\varepsilon^{\prime \prime }\geq 0$, that is, if the wires are passive or lossy.

## 3. Analytical regularization based on single-wire part inversion

To reduce the DR problem to a well-conditioned algebraic equation, we represent the total magnetic field as follows (here and further, the index *z* is omitted):


(1.3) $$H^{\text{tot}}\left(r,\varphi \right)=\left\{ \begin{array}{l} H^{\text{int}(p)}\left(r,\varphi \right),\;\;\;\left(r,\varphi \right)\in \left\{1.p\right\},\;p=1...M,\\ H_{,}^{0}\left(r,\varphi \right)+H^{\text{ext}}\left(r,\varphi \right),\;\;\left(r,\varphi \right)\in \left\{2\right\},\; \end{array}\right.$$

and expand it in terms of the Fourier series of the azimuth harmonics, with bounded coefficients, inside each of domains {1 .*p*} and as a superposition of *M* similar to outgoing expansions in domain {2}, respectively,


(1.4) $$H^{\text{int}\left(p\right)}\left(r,\varphi \right)=\sum_{n=-\infty }^{\infty }y_{n}^{\left(p\right)}J_{n}\left(k\alpha r_{p}\right)e^{in\varphi_{p}},\;\;\left(r_{p},\varphi_{p}\right)\in \left\{1.p\right\},$$

(1.5) $$\begin{array}{rl} & H^{0}(r_{p},\varphi_{p})\\ & \;=A\beta e^{ikx_{p}/\beta }\sum_{n=-\infty }^{\infty }i^{n}\beta^{-n}\left\{\begin{array}{ll} -e^{q(y_{p}-h)}(1-\gamma {)}^{n}, & p=1,\ldots ,M_{1}\\ e^{-q(y_{p}-h)}(1-\gamma {)}^{n}, & p=M_{1}+1,\ldots ,M \end{array}\right\}J_{n}(kr_{p})e^{in\varphi_{p}},\;(r_{p},\varphi_{p})\in \{2\}, \end{array}$$

(1.6) $$H^{\text{ext}}\left(r,\varphi \right)=\sum_{q=1}^{M}\sum_{n=-\infty }^{\infty }x_{n}^{\left(q\right)}{\overset{˜}{w}}_{n}H_{n}^{\left(1\right)}\left(kr_{q}\right)e^{in\varphi_{q}},\;\;\;\left(r,\varphi \right)\in \left\{2\right\},$$

where $x_{n}^{\left(p\right)},y_{n}^{\left(p\right)}$ are unknown coefficients, $H_{n}^{\left(1\right)}(\cdot )$ and $J_{n}(\cdot )$ are the first-kind Hankel and Bessel functions, respectively, and ${\tilde{w}}_{n}$ is a certain weight specified further to obtain well-conditioned matrix equation.

Note that, equation (1.5) is derived from equation (1.1) using the Anger formula (see [5]), and the series of equations (1.4)–(1.6) satisfy the Helmholtz equations, the condition of the local power finiteness and the radiation condition, respectively. To determine the unknown expansion coefficients, these series are substituted into the boundary conditions equation (1.2) at the contours of the wires. For the term $H_{n}^{\left(1\right)}(kr_{q})e^{in\phi_{q}}$ with q≠p, arising from equation (1.6), Graf’s addition theorem is applied as in [27, equation (3)].

Further, we perform analytical inversion of the single-wire parts of each of *M* blocks of the obtained equation. This is done by multiplying the *m*th equation with $\exp(-im^{\prime }\varphi )$, integrating it in φ from zero to 2*π*, and using the orthogonality of exponents. After the exclusion of coefficients $y_{n}^{\left(p\right)}$, we obtain a block type (*M* × *M*) infinite-matrix equation for the remaining coefficients. Omitting the superscript of the Hankel function and using the prime to mark the differentiation in argument, we get


(1.7) $$x_{m}^{\left(p\right)}+\frac{V_{m}}{{\overset{˜}{w}}_{m}D_{m}}\sum_{\begin{array}{c} j=1\\ j\neq p \end{array}}^{M}\sum_{n=-\infty }^{+\infty }{\overset{˜}{w}}_{n}x_{n}^{\left(j\right)}H_{m-n}\left(kL_{pj}\right)e^{i\left(n-m\right)\psi_{pj}}=\frac{F_{m}^{\left(p\right)}}{{\overset{˜}{w}}_{m}D_{m}},$$

where


(1.8) $$V_{m}^{,}=iZ^{-1}J_{m}^{\prime }\left(ka\right)J_{m}^{\prime }\left(k\alpha a\right)+\alpha J_{m}^{\prime }\left(ka\right)J_{m}\left(k\alpha a\right)-J_{m}\left(ka\right)J_{m}^{\prime }\left(k\alpha a\right),$$

(1.9) $$D_{m}=iZ^{-1}H_{m}^{\prime }\left(ka\right)J_{m}^{\prime }\left(k\alpha a\right)+\alpha H_{m}^{\prime }\left(ka\right)J_{m}\left(k\alpha a\right)-H_{m}\left(ka\right)J_{m}^{\prime }\left(k\alpha a\right),$$

(1.10) $$F_{m}^{\left(p\right)}=-iZ^{-1}f_{m}^{\prime \left(p\right)}J_{m}^{\prime }\left(k\alpha a\right)-f_{m}^{\prime \left(p\right)}\alpha J_{m}\left(k\alpha a\right)+f_{m}^{\left(p\right)}J_{m}^{\prime }\left(k\alpha a\right),$$

where the incident field caused quantities are


(1.11) fm(p)=fm(p)(ka)=Aeikxp/βimJm(ka)β1−m{−eq(yp−h),p=1,…,M1,−e−q(yp−h),p=M1+1,…,M.

Note that, if there is a single wire, *M* = 1, then the summations in equation (1.7) vanish, and this equation turns into an explicit expression for the unknowns, $x_{m}^{\left(p\right)}=F_{m}^{\left(p\right)}D_{m}^{\;-1}\tilde{w}{\;}_{m}^{-1}$, as expected due to the single-wire part inversion. Now, if the weight coefficients are taken as large-index asymptotics of the Bessel functions $J_{n}(ka)$, i.e. as [28], then


(1.12) $${\overset{˜}{w}}_{n\geq 0}={\left(ka/2\right)}^{n}/n!,\;{\overset{˜}{w}}_{n<0}={\left(-1\right)}^{n}{\overset{˜}{w}}_{n>0},$$

and the obtained infinite-matrix equation (1.7) is of the Fredholm second kind in the space of number sequences $l_{2}^{M}$, provided that for all *p* and *j*, $L_{pj}>2a$, i.e. the wire contours do not touch one another (see appendix).

Here, it should be noted that, the algebraic equations, similar to equation (1.7); however, without the weight equation (1.12), were first time derived for the plane-wave scattering in [18] and then reproduced in many other publications, e.g. [19–23], including a recent tutorial [24]. Such equations, unfortunately, do not amend truncation because their matrix elements decay exponentially with one index, they grow, however, exponentially with the other index. This defect can be fixed by introducing the weight, $J_{n}(ka)$, as found in [29–31]. Still, such a weight brings additional singularities into the matrix elements, at the real-valued zeros of the Bessel functions. In computations, these singularities are usually not felt because the cylindrical functions are easily computed with machine precision; however, the choice of the weight as equation (1.12) eliminates that defect entirely.

## 4. Plasmon mode characterization via the Kubo formalism

The most widely adopted model of the electron mobility in the graphene monolayer is the Kubo formalism [11]. Here, the graphene thickness is considered zero, and the hexagonal fine structure of graphene is neglected. Under these assumptions, which are justified up to the X-ray frequencies, graphene’s surface conductivity, $\sigma (\omega ,\mu_{c},\tau ,T)$ depends on the cyclic frequency *ω*, chemical potential *μ_c_*, electron relaxation time *τ* and temperature *T*. Its value consists of two contributions, $\sigma =\sigma_{\text{intra}}+\sigma_{\text{inter}}$, which are the intraband and interband conductivities. That is,


(1.13) $$\sigma_{\text{intra}}=-\frac{i\;\Omega \;Z_{0}}{\omega +i\tau^{-1}},\;\Omega =\frac{q_{e}^{2}k_{B}T}{\pi \hbar^{2}Z_{0}}\left\{\frac{\mu_{c}}{k_{B}T}+2\ln\left[1+\exp\left(-\frac{\mu_{c}}{k_{B}T}\right)\right]\right\},$$

where $Z_{0}=\sqrt{\mu_{0}/\varepsilon_{0}}$ is the impedance of the free space, and $\sigma_{\text{inter}}$ is expressed as an integral of known functions (see [11]). The normalized surface impedance (or resistivity) of graphene is $Z(\omega )=Z_{0}^{\;-1}{\left(\sigma_{\text{intra}}+\sigma_{\text{inter}}\right)}^{\;-1}$.

The relative contribution of the two terms in *Z* depends on the frequency and chemical potential [11,32,33], so that at $\mu_{c}=1\text{ eV}$, $\sigma_{\text{inter}}$ is less than 0.001 of $\sigma_{\text{intra}}$, in absolute value, if the frequency is below 60 THz. This allows us to derive approximate expressions for the characteristics of the transversal plasmon natural modes on graphene-coated wire [32,33]. Their complex wavenumbers are the roots of the transcendental equations, $D_{m}(k,a,\varepsilon ,\Omega )=0$, *m* = 0,1,2,.. which are located in the quasi-static domain, where ka<<1. That is, the real parts of their frequencies, $f_{m}^{P}=k_{m}^{P}c/2\pi$ and the Q-factors are approximately found as follows (*m* = 1,2,3,…):


(1.14) $$f_{m}^{P}\approx \frac{1}{2\pi }{\left[\frac{mc\Omega }{a\left(\varepsilon +1\right)}\right]}^{1/2},\;\;Q_{m}^{P}\approx \frac{4\varepsilon \tau }{{\left(1+\varepsilon \right)}^{3/2}}{\left(\frac{mc\Omega }{a}\right)}^{1/2}.$$

Note that, in addition to the plasmon modes, there is another class of zeros of the same $D_{m}$—these are the modes of the circular dielectric wire, perturbed by the presence of graphene cover [32]. The frequencies of these modes, however, are located in the higher frequency domain starting from, approximately, $c/4a\sqrt{\varepsilon }$.

## 5. Nanowire dimer, beam between wires

Using equations (1.7)–(1.12), we analyse the DR effect for a dimer of circular dielectric nanowires wrapped in graphene with the electron beam moving between the wires, so that *M*_1_ = 1, *M* = 2 and $x_{1,2}=0,\;y_{1,2}=a\pm s/2$.

**Figure 2.**
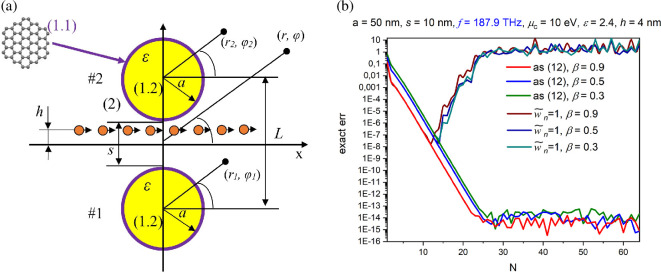
Cross-section of two graphene-covered dielectric wires and electron beam moving between them (a) and the computational error as a function of the matrix block truncation number, *N* (b).

In figure 2*b*, we demonstrate the computational error, by the *l*_2_-norm (see appendix for the error definition), of the numerical solution of equation (1.7) as a function of the block truncation order, *N*. As one can see, although the nanowires are strongly coupled as 2 a*/L* = 0.909, the error goes down exponentially all the way to machine precision, reached with *N* less than 30. A reasonable accuracy of four digits is achieved with 15 coefficients in the local Fourier series (*n* = 7), although this value increases if the beam velocity *β* gets smaller—because of the less quick decrement of the right-hand part elements of equation (1.7). For comparison, the error without the weight equation (1.12) demonstrates catastrophic behaviour at larger *N*.

**Figure 3.**
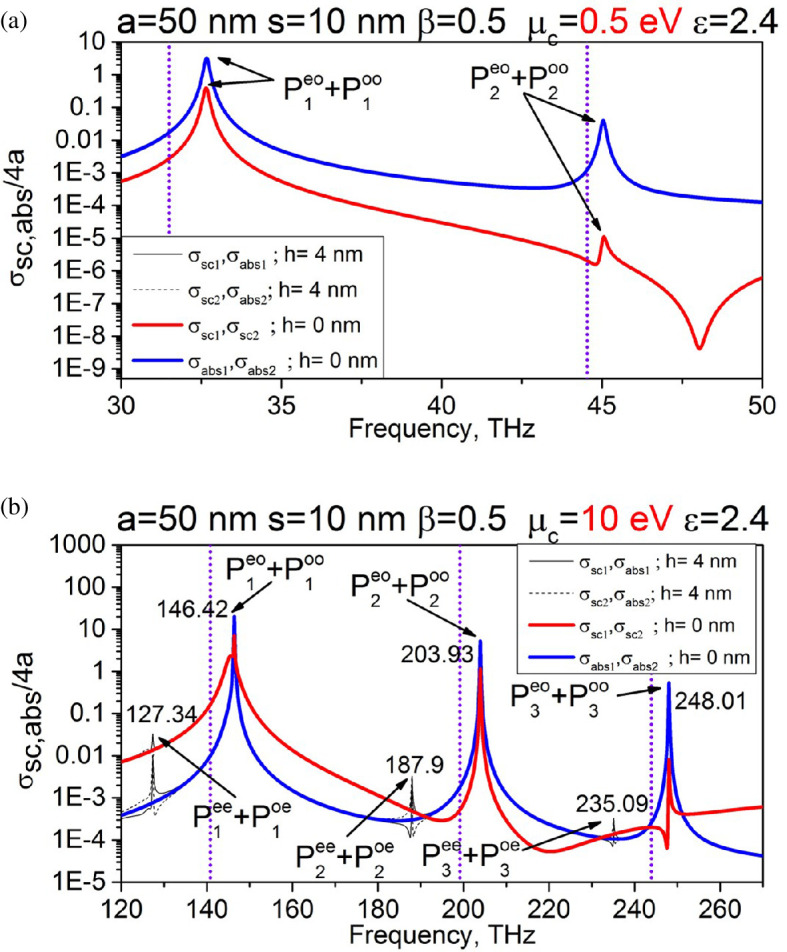
Normalized partial SCS and ACS versus the frequency for nanowire dimer excited by the electron beam moving between wires. Wire radius is *a* = 50 nm, air gap width is *s* = 10 nm, chemical potential is *μ*_c_ = 0.5 eV (a) and 10 eV (b), and beam shift is *h* = 0 and 4 nm. Vertical dotted lines correspond to equation (1.16).

Figure 3 presents the normalized partial scattering and absorption cross-sections (SCS and ACS, see [33]) versus the frequency for the wires with *a* = 50 nm, *s* = 10 nm, beam velocity *β* = 0.5; graphene parameters are *T* = 300 K, *τ* = 1 ps, chemical potential *μ_c_* = 0.5 eV (a) and 10 eV (b). These plots prove that the considered configuration can serve, under certain conditions, as a model of the nanosize BPM [33].

Indeed, one can see that the DR-induced radiated and absorbed powers demonstrate resonance behaviour. As explained in [33], these resonances are caused by the excitation of the natural modes of the dimer as an open resonator. Because of twofold symmetry, these modes are ‘supermodes’ in the sense explained in [33,34].

As seen in panel (a), the plots for *μ*_c_ = 0.5 eV do not demonstrate any effect of the shift of the beam trajectory. The curves for *h* = 0 and *h* = 4 nm overlap and reveal only the resonances on the *y*-odd plasmon supermodes, $P_{1,2}^{EO}$and $P_{1,2}^{OO}$, which stay unresolved because of low *Q*-factors [33,34].

According to equation (1.14), to enhance the *Q*-factors, either a larger chemical potential or a smaller wire radius is needed. Indeed, if *μ*_c_ = 10 eV, then the SCS and ACS plots for the shifted beam demonstrate new high-*Q* resonances, which are not present if the beam passes through the midpoint of the air gap. New peaks correspond to the unresolved supermodes $P_{1,2,3}^{EE}$ and $P_{1,2,3}^{OE}$ with the fields, orthogonal to the beam field equation (1.2) at *h* = 0.

As expected, the *y*-even and *y*-odd supermode frequencies are shifted in opposite manner from the single-wire frequency equation (1.14), indicated by the vertical dotted lines. This is exactly the effect, which is needed for the beam monitoring (first reported in [33]). Here, we note that, its counterpart exists in the visible-light DR originating from beam-excited high-permittivity dielectric nanowire dimers and silver nanotube dimers [35,36].

**Figure 4.**
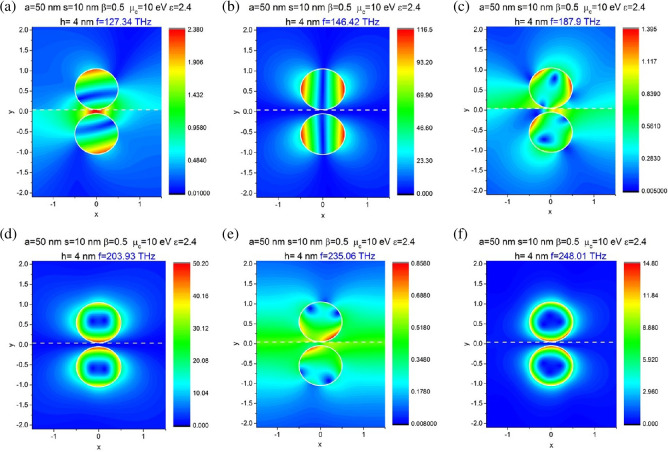
In-resonance near magnetic field patterns of graphene-covered dielectric nanowire dimer with wire radius *a* = 50 nm, the chemical potential *μ*_c_ = 10 eV, beam shift *h* = 0 and 4 nm and air gap of *s* = 10 nm.

Figure 4 presents the nearfield patterns computed in the plasmon resonances for the beam, shifted by *h* = 4 m. They show the features, which agree with interpretation presented above.

## 6. Finite nanowire array, beam over wires

**Figure 5.**
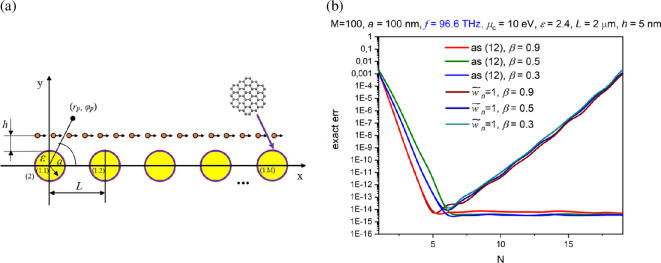
Cross-section of array of *M* graphene-covered dielectric wires and electron beam moving over them (a) and the computational error as a function of the matrix block truncation number (b).

In this section, we study DR effect for the configuration shown in figure 5. This is a finite-periodic array made of *M* identical graphene-covered circular dielectric wires. It is excited by the modulated electron beam field equation (1.1).

In figure 5*b*, we plot the computational error, by the *l*_2_-norm (see appendix A), of the numerical solution of equation (1.7) as a function of the block truncation order, *N*, for the array of *M* = 100 nanowires of radius *a* = 10 nm and period *L* = 2 μm. As in the previous case, the error goes down exponentially all the way to machine precision. Still, the accuracy of 3−4 digits is achieved already with *n* = 1, i.e. with zeroth and plus or minus first coefficients in the local Fourier series—this is because here the sub-wavelength wires (*a* ≈ *λ*/50) are very far from each other, 2*a/L* = 0.001, and their optical interaction is very weak. The machine precision is reached, in this case, with less than 15 Fourier coefficients (*n* = 7) although this value increases if the beam velocity *β* gets smaller. Similar to the previous section, the error for the code without the weight equation (1.12) displays numerical ‘explosion’ if *N* exceeds certain value.

**Figure 6.**
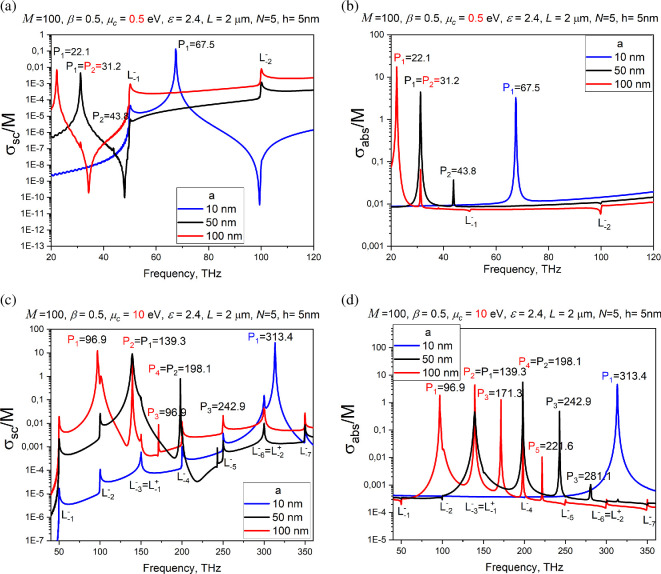
Normalized DR TSCS versus the frequency for the grating of *M* = 100 graphene-covered dielectric wires of radii *a* = 10, 50 and 100 nm and the chemical potential *μ_c_* = 0.5 eV (a) and 10 eV (b).

This configuration can serve as a model of the DLA section [9,10,27]. Figure 6 shows the spectra of per-wire total SCS (TSCS) for the *M* = 100, array period *L* = 2 μm and chemical potential *μ_c_*c= 0.5 eV (a) and 10 eV (b).

In the depicted frequency range, we can identify the resonances on the plasmon modes *P_m_* and the lattice modes *L_m_*. Note that, the plasmon-mode resonance displays only one peak, in contrast to the dimer-mode quartets found in the previous section. This is because here the wires are so far from each other (2*a/L* ≤ 0.1) that the supermodes are not resolved. The lattice-mode resonances are very close (from the red side) to the frequencies of the Rayleigh anomalies of the corresponding infinite grating of similar wires, given by the equation, , *m* = 0, 1, 2, … [37–39].

Investigating the influence of the wire radius on the grating modes, we compare the results for *a* = 10, 50 and 100 nm. In line with [11], the plasmon-mode frequencies scale as , while the lattice modes stay fixed.

**Figure 7.**
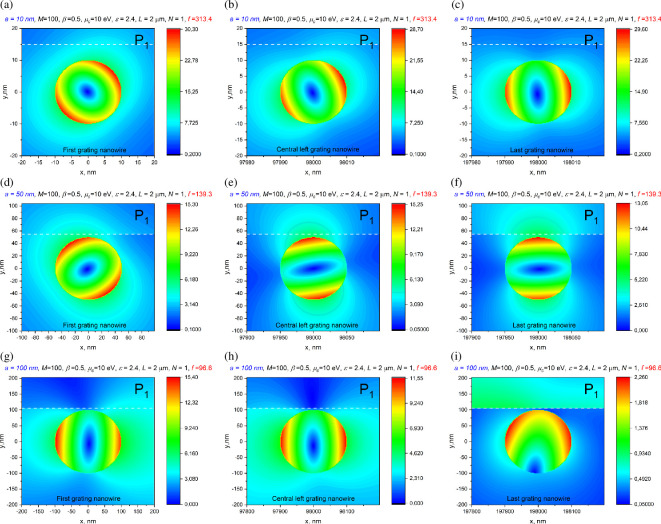
The near magnetic field patterns in the *P*_1_ plasmon-mode resonances marked in figure 6*b*, near the first, the 49th and the 100th wire.

In figure 7, we present the near magnetic field patterns for the grating of figure 9, in the resonance on the *P*_1_ mode, near the first, the central-left and the last nanowire. The *P*_1_ pattern is better visible in the case of the smallest wire radius, because here the *Q*-factor of *P*_1_ is the largest.

## 7. Conclusions

We have presented basic equations and numerical results for the DR of a modulated electron beam in the presence of a dimer and a finite array of identical circular dielectric graphene-covered nanowires. Assuming that the particle velocity and trajectory are fixed, we have treated this phenomenon as a classical wave-scattering problem, where the properties of graphene are accounted for with the aid of quantum-theory description of its conductivity. Using the single-wire part inversion, we have reduced that problem to a Fredholm second kind matrix equation that guarantees the solution convergence and enables easy control of computational accuracy.

For the closely spaced nanowire dimer, we have demonstrated the appearance of additional resonances on the plasmon supermodes of the graphene covers, excited only if the beam is shifted from the symmetrical trajectory.

For the sparse finite array of nanowires, we have demonstrated the presence of the plasmon-mode resonances of each wire and the other, collective resonances on the lattice modes, caused by the periodicity (that can be also understood via the Bragg effect). This analysis can be useful in the design of BPMs and DLA sections, respectively, made of low-index dielectric wires coated with graphene.

## Figures and Tables

**Figure 8.**
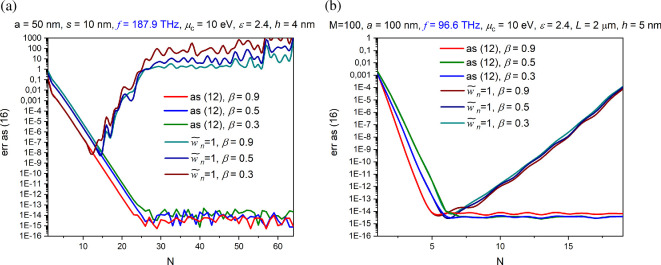
The computational error with respect to the reference solution as a function of the matrix block truncation number, *N*, for the dimer configuration of figure 2*b* (a) and the grating configuration of figure 5*b* (b).

## Data Availability

All computations relating to the results presented in this paper can be readily reproduced by a reader by using the equations explicitly provided in the paper.

## Appendix A.

The Fredholm second-kind nature of the matrix equation (1.7) follows from the estimations of the necessary conditions, that are valid for all *p*, *j* = 1, …, *M*,

(A 1)
$$\begin{array}{r} \sum_{m,n=-\infty }^{+\infty }|\frac{{\overset{˜}{w}}_{n}V_{m}}{{\overset{˜}{w}}_{m}D_{m}}H_{m-n}\left(kL_{pj}\right){|}^{\;2}<\text{const}\cdot \sum_{m,n=0}^{+\infty }{\left(\frac{2a}{L_{pj}}\right)}^{m+n},\\ \;\sum_{m=0}^{+\infty }|\frac{F_{m}^{\left(p\right)}}{{\overset{˜}{w}}_{m}D_{m}}{|}^{\;2}<\;\text{const}\cdot e^{-q|y_{p}-h|}\sum_{m=0}^{+\infty }\frac{1}{m!}{\left(\frac{ka}{2\beta }\right)}^{m}, \end{array}$$

which are obtained after replacing the cylindrical functions with the first terms of their power series representations.

∗∗∗

The computational error in the solution of the matrix equation (1.7), shown in figures 3*b* and 6*b*, is defined as the difference, in *l*_2_-norm, between two adjacent solutions, i.e. computed with each block truncated by the orders *n* + 1 and *N*,

(A 2)
$$error\left(N\right)=\|{\vec{x}}_{N+1}-{\vec{x}}_{N}\|/\|{\vec{x}}_{N+1}\|.$$

It is interesting to see that the errors in ${\vec{x}}_{N}$ with respect to the reference result, ${\vec{x}}_{ref}$, which is assumed as the result, computed by our code with the maximum truncation number, *n* = 65 and *n* = 20, respectively, demonstrate very similar dependences on *N* (see figure 8).
